# Impact of imprecise household location on effective coverage estimates generated through linking household and health provider data by geographic proximity: a simulation study

**DOI:** 10.1186/s12942-021-00292-y

**Published:** 2021-08-21

**Authors:** Emily D. Carter, Melinda K. Munos

**Affiliations:** Institute for International Programs, Johns Hopkins Bloomberg School of Public Health, 615 North Wolfe Street, Baltimore, MD USA

**Keywords:** GIS, Quality-adjusted coverage, Linking, Household survey, Facility assessment, Research methods

## Abstract

**Background:**

Geographic proximity is often used to link household and health provider data to estimate effective coverage of health interventions. Existing household surveys often provide displaced data on the central point within household clusters rather than household location. This may introduce error into analyses based on the distance between households and providers.

**Methods:**

We assessed the effect of imprecise household location on quality-adjusted effective coverage of child curative services estimated by linking sick children to providers based on geographic proximity. We used data on care-seeking for child illness and health provider quality in Southern Province, Zambia. The dataset included the location of respondent households, a census of providers, and data on the exact outlets utilized by sick children included in the study. We displaced the central point of each household cluster point five times. We calculated quality-adjusted coverage by assigning each sick child to a provider’s care based on three measures of geographic proximity (Euclidean distance, travel time, and geographic radius) from the household location, cluster point, and displaced cluster locations. We compared the estimates of quality-adjusted coverage to each other and estimates using each sick child’s true source of care. We performed sensitivity analyses with simulated preferential care-seeking from higher-quality providers and randomly generated provider quality scores.

**Results:**

Fewer children were linked to their true source of care using cluster locations than household locations. Effective coverage estimates produced using undisplaced or displaced cluster points did not vary significantly from estimates produced using household location data or each sick child’s true source of care. However, the sensitivity analyses simulating greater variability in provider quality showed bias in effective coverage estimates produced with the geographic radius and travel time method using imprecise location data in some scenarios.

**Conclusions:**

Use of undisplaced or displaced cluster location reduced the proportion of children that linked to their true source of care. In settings with minimal variability in quality within provider categories, the impact on effective coverage estimates is limited. However, use of imprecise household location and choice of geographic linking method can bias estimates in areas with high variability in provider quality or preferential care-seeking.

## Background

Combining data from household and health facility assessments can be used to estimate effective coverage of essential health service settings [1] and assess barriers to improved population health. Data from household surveys [such as the Demographic and Health Survey (DHS) and Multiple Indicator Cluster Survey (MICS)] provide a population-based denominator of intervention need and care-seeking for services. Health provider assessments [such as the Service Provision Assessment (SPA) and Service Availability and Readiness Assessment (SARA)] offer information on provider quality, including structural quality and potentially provision of care. Linking these two data sources can be used to estimate effective coverage, or the proportion of the population in need of a service that received it with sufficient quality to achieve a health benefit. These estimates provide a more complete picture of the care likely received by a population, for example, the proportion of women who delivered at a health facility with sufficient structural resources and competence to provide appropriate labor and delivery care. However, the methods used for combining data sets and aspects of the data sources can influence results.

Various methods for linking household and provider data exist [2]. Linking at the ecological level is the most common approach and includes assigning an individual to one or more providers based on geographic proximity or administrative catchment area [2]. This method is often used as existing household surveys ask about the type of provider utilized but not the specific name of the provider or facility. Ecological linking assumes geographic access is the driving force in determining source of care.

Current household surveys typically collect imprecise household location data, potentially introducing bias into analyses based on geographic proximity. Demographic and Health Surveys collect data on a single central population point within a sampling cluster, or enumeration area, rather than the location of individual households. The DHS randomly displaces the cluster central point to preserve respondent confidentiality [3]. Multiple Indicator Cluster Surveys do not collect GIS data regularly and refer data users to contact country statistics offices to access cluster locations mapped in census cartography [4]. Guidelines on the use of DHS GPS data note that use of displaced DHS location data can increase the bias and error for analyses using the distance between clusters and resources as a covariate [5].

A previous analysis assessed the amount of bias introduced to estimates of effective coverage of child curative services generated using different ecological linking methods against estimates generated using data on true sources of care for a population in Southern Province, Zambia [6]. Carter and colleagues found most ecological linking methods produced statistically equivalent estimates when conditioning the ecological linking on type of provider from which care was sought for the illness. However, those ecological linking analyses which employed measures of geographic proximity used data on the exact location of each sick child’s primary residence.

Using data on care-seeking for child illness and health provider quality in Southern Province, Zambia, we assessed the potential error introduced to effective coverage linking analyses by using original and displaced cluster central point location in place of household location. We assessed the proportion of children linked to their true source of care using original and displaced cluster locations and compared estimates of quality-adjusted coverage of curative child health services generated using measures of geographic proximity based on household location, undisplaced cluster location, and displaced cluster location to gauge bias in estimates.

## Methods

### Study design, data collection, and key measures

We performed a secondary analysis of data collected in Southern Province, Zambia as part of a study assessing the feasibility and performance of exact-match and various ecological linking methods. A detailed description of the study methods and findings has been published previously [6]. Briefly, we conducted the study in five health facility catchment areas in Choma district between January and March 2016. The study collected data on care-seeking for illness in children under 5 (fever, diarrhea, or suspected ARI) in the preceding 2 weeks, using a household survey instrument based on the Zambia DHS. In addition to the standard DHS questions on the type of provider from which care was sought for reported child illness, we also asked mothers to identify (name or describe) the specific source(s) of care utilized. We also collected data on structural quality, or infrastructure required, for managing child illness for every health care provider in the study area using questions derived from the Service Availability and Readiness Assessment (SARA). The structural quality indicators were designed to assess a provider or facility’s capacity to provide curative services for children, including the presence of drugs and commodities, training, supervision, and provider case management knowledge. We included public, private, informal, and traditional sources of care in the assessment. Geo-locations of all participating households and health care providers were collected using the geopoint function built into Open Data Kit (ODK) Collect.

### Analysis

Overall approach We used data generated using the exact-match approach as the measure of true quality-adjusted effective coverage of management of child illness in the study population. Using the exact-match linking approach, we assigned each child the structural quality score of the specific provider from which care was reportedly sought, which was considered to be the true source(s) of care. Children were not linked using the exact-match method if their caregiver could not recall the name of the provider or facility from which care was sought or the provider could not be located for inclusion in the study, mostly affecting individuals who utilized informal shops.

To simulate ecological linking in the absence of data on specific source of care, each sick child was linked to the closest health provider(s) within the reported category of source of care (Box 1) using three measures of geographic proximity: (1) Euclidean distance, (2) travel time, and (3) 5 km radius. Each measure of geographic proximity was applied using (1) known household location, (2) undisplaced cluster location, and (3) five sets of displaced cluster locations, each reported separately. Quality-adjusted coverage of management of child illness was calculated using each combination of ecological linking method and measure of sick child location by assigning each child the quality score of the proximal provider(s) to which they were linked.

**Table:** 

Box 1. Categories of healthcare providers in the study area
Public
Government hospital
Government health center/post
Government CBA/fieldworker
Private
Private hospital/clinic
Pharmacy
Informal
Shop/market
Traditional/faith-based practitioner

For both the exact-match and each ecological linking approach, we calculated the quality-adjusted coverage of management of child illness as the average quality score across all sick children in the study. If no care was sought for a sick child, they were assigned a quality score of zero. If care was sought from multiple sources, we averaged the scores of those sources. If a child could not be linked to a provider using exact source or geographic proximity, they were assigned the average score for the provider category.

To quantify the bias introduced into each method by imprecise household location, we compared the estimates of quality-adjusted coverage from each combination of ecological linking approach and cluster location against the (1) exact-match quality-adjusted coverage estimates and (2) estimates generated using each ecological approach with the true household location. We also assessed how accurately each approach identified the actual provider(s) utilized by each sick child by comparing the provider(s) linked to each sick child using the ecological approaches with the specific source(s) of care reported by each child’s mother.

Quality score A full description of the construction of provider structural quality scores and methods for defining geographic proximity are presented in a previous publication [6]. Briefly, we defined each provider’s structural quality score as the availability of services, commodities, and human resources needed to appropriately manage common child illnesses (Box 2). These indicators were considered the minimum inputs for appropriate care: the basic commodities required to diagnose and treat common child illness, along with the human resources and clinical knowledge to apply them correctly. As such, the score reflects an upper ceiling of the potential quality of care offered by a provider. A provider received one point for each indicator if requirements were met and zero if not; each domain (bold italicized in Box 2) received equal weight. We calculated scores as a continuous variable ranging from zero (no capacity to provide care) to 100% (full capacity to provide care).

**Table:** 

Box 2. Structural quality score components			
***Diagnostics***			
Malaria diagnostic (RDTs or microscopy)			
Malnutrition diagnostic (MUAC or scale + height board + growth chart)			
ARI diagnostic (stethoscope or respiratory timer)			
General microscopy (functioning microscope and slides)			
***Basic medicines***			
ORS			
Zinc			
ACT			
Oral antibiotic			
***Severe/complicated illness medicines***			
IV fluids			
Injectable quinine or artesunate			
Injectable antibiotics			
***Human resources***			
Training (at least one staff member with IMCI or relevant training)			
Guidelines (IMCI guidelines or relevant guidelines or job aid available)			
Supervision (received supervision visit with case management observation in past 3 months)			
***Available services***			
Diagnosis and treat malaria (by pathology)			
Diagnosis and treat diarrhea (by pathology)			
Diagnosis and treat ARI (by pathology)			
Diagnosis and treat malnutrition (by pathology)			
Facilitated referral capacity			
***Knowledge***			
Average health worker performance on 4 clinical case scenarios

#### Geographic proximity

We employed three measures of geographic proximity in this analysis. For each method, we developed an automated script in QGIS comparable to the process outlined for application in ArcGIS in a previous paper [6]. We conducted all geographic analyses in QGIS 2.18.24 (Open Source Geospatial Foundation Project, Beaverton, OR, USA). Ecological linking was restricted to only assign children to the types of providers (managing authority and level of care) from which care was reportedly sought based on responses during the household survey. For example, if a mother reported care for her sick child from a government health center, then the child could only be linked to another government health center—not a private facility or a government hospital.

- Euclidean distance: each sick child was linked to the single closest provider based on Euclidean distance from the child’s location within the reported source of care provider category. This method is the simplest approach for assigning a child to a specific provider.
- Travel time: each sick child was linked to the single closest provider by travel time from the child’s location within the reported source of care provider category. Travel time was approximated by grading the relative speed of travel on different types of roads (e.g., paved roads, graded roads, footpaths). Data on road networks were derived from Open Street Maps (OSM) and local expertise where absent in OSM. This method is designed to model the effect of road access and quality on care-seeking.
- 5 km radius: each sick child was linked to all providers within the source of care provider category within a 5 km radius of the child’s location. This method is designed to approximate a 1-hour walking distance from a home to a provider in any direction.

#### Cluster location and displacement

The central point location for each cluster was generated to capture an area of high population density within each cluster inline the DHS central point measurement procedures. A census of all households within each of the study catchment areas was conducted before the study and included the location of each household. In QGIS, we grouped all the households into clusters of 150 households based on measured latitude and longitude, and we calculated the mean point of each cluster of 150 households as the central point.

Each central point was displaced five times using an R script developed by Measure Evaluation for DHS cluster displacement [5]. In brief, the code offsets each point using a random angle and random distance, capped at 5 km for rural clusters (1% capped at 10 km) and 2 km for urban clusters. The code further restricts the displacement to ensure points are not displaced outside of their true administrative unit (e.g., district). However, this feature was redundant in our analysis due to the small size of the study area. We ran the displacement code in R 3.4.3 (R Foundation for Statistical Computing, Vienna, Austria) and we imported each set of displaced coordinates into QGIS for the linking analyses.

**Fig. 1:**
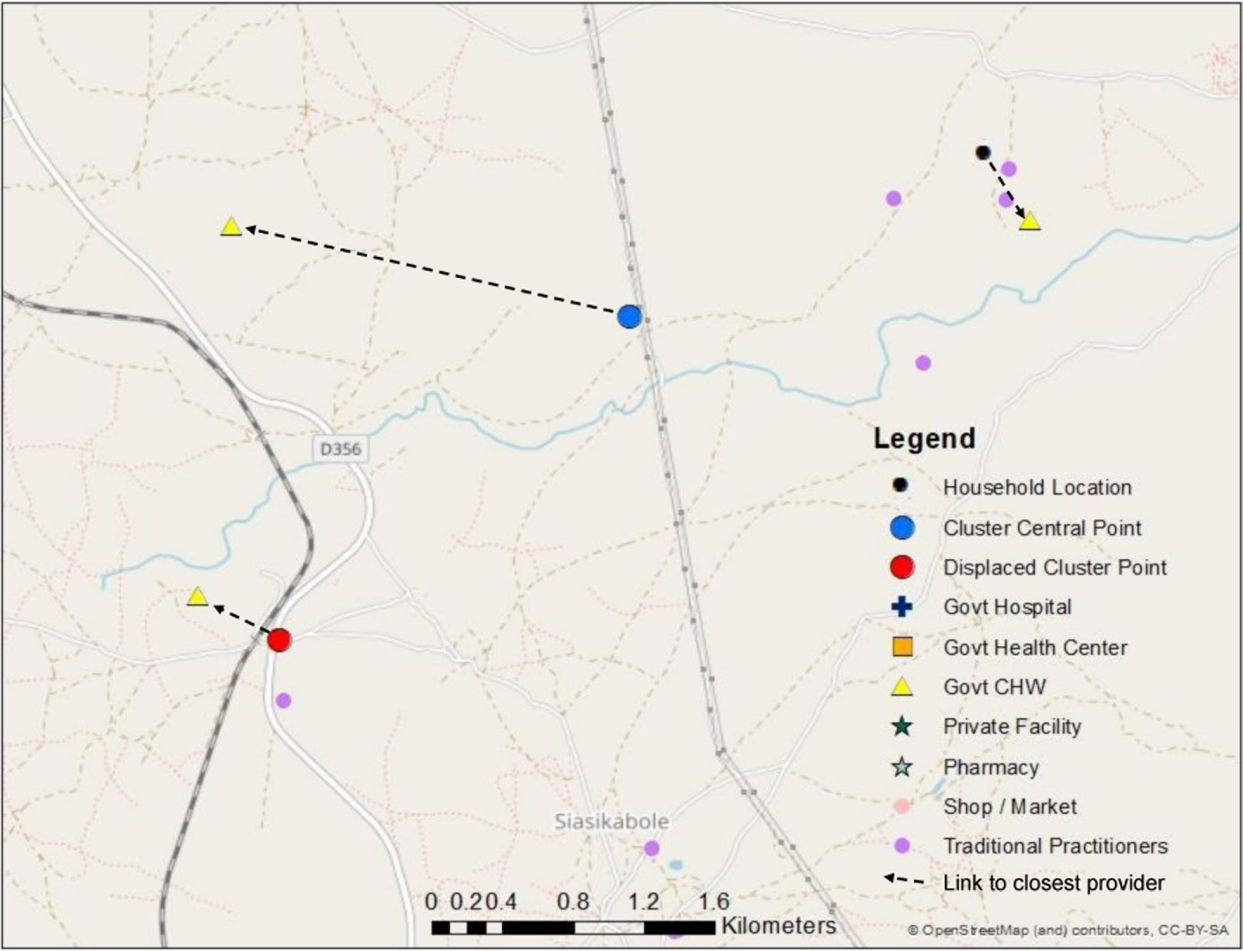
Map of link to closest CHW based on true household location, cluster central point, and displaced cluster point

We then substituted each central point and displaced central point for the household location in our measures of geographic proximity. Instead of calculating the geographic proximity of providers from the home of each sick child, we measured proximity from the relevant central point or displaced central point location as depicted in Fig. 1.

#### Sensitivity analysis

In our dataset, there was limited variability in quality within provider categories. This limited the potential generalizability of our simulation results. Therefore, we ran two additional sensitivity analyses using simulated quality scores to assess the effect of sampling in settings with greater diversity in quality scores.

##### Quality simulation 1: preferential care-seeking from higher-quality facilities

In the first simulation, we maintained the data on household care-seeking behavior as well as facility, household, and cluster locations (displaced and undisplaced) from the primary analysis. However, each facility was assigned a structural quality score designed to simulate preferential care-seeking in favor of higher-quality facilities within a provider category. The rest of the of the effective coverage estimation methods were implemented in the same manner as in the above section. Facilities that were utilized more frequently based on the household survey data were given higher quality scores than those that were utilized less frequently or not at all. We calculated how many times a respondent reported utilizing a specific source of care. We then ranked each provider within a provider category based on utilization. If a provider saw more than the median number of reported visits within the category, we increased the provider quality score by two standard deviations of the overall provider category quality score. If a provider saw none of the sampled children or fewer than the median number of reported visits within the category, we decreased the provider quality score by two standard deviations of provider category score. This resulted in a data set in which caregivers more frequently accessed care from higher quality health providers, simulating a setting of selective bypassing of lower-quality providers within a given level of care.

##### Quality simulation 2: random quality

In our second simulation, as in the previous simulation, we maintained the data on location and household care-seeking behavior. However, each facility was assigned a structural quality score completely at random. The rest of the effective coverage estimation methods were implemented in the same manner as in the above section.

## Results

A full description of the study population, loss to follow-up, and healthcare provider characteristics are available in a previous publication [6]. Three hundred and thirty five rural and 469 urban households with at least one child under 5 were enrolled in the study. 7.1% of households were lost to follow-up prior to administration of the household care-seeking interview. Among the 1084 children included in the household care-seeking survey, 35% of urban children and 36% of rural children experienced at least one illness meeting DHS criteria in the 2 weeks preceding the survey, primarily fever (Table 1). Most mothers (79% rural; 67% urban) reported seeking care for their child’s illness. Most children sought care from a skilled provider, including government health facilities, government community-based agents (CBAs), and private clinics. Government health centers were the primary reported source of care in both the urban (60%) and rural (61%) areas. In the rural area, 18% of children were taken to a CBA for care. In the urban area, care was sought for 5% of children from informal shops. Hospitals, pharmacies, private facilities, and traditional practitioners accounted for a small number of care-seeking events.

**Table 1:** Characteristics of reported child illness and care-seeking events, by stratum

	Rural			Urban		
	n 547	%	CI	n 537	%	CI
Proportion of children with at least one DHS illness	199	36.4	[32.4–40.5]	186	34.6	[30.7–38.8]
Reported Child Illness	199			186		
Diarrhea	23	11.6	[7.8–16.8]	50	26.9	[21.0–33.7]
Fever	117	58.8	[51.8–65.4]	85	45.7	[38.7–52.9]
ARI^a^	6	3	[1.4–6.6]	3	1.6	[0.5–4.9]
Diarrhea and Fever	28	14.1	[9.9–19.6]	35	18.8	[13.8–25.1]
Diarrhea and ARI	3	1.5	[0.5–4.6]	0	0	–
Fever and ARI	17	8.5	[5.4–13.3]	10	5.4	[2.9–9.7]
Diarrhea, Fever and ARI	5	2.5	[1.0–5.9]	3	1.6	[0.5–4.9]
Proportion of illnesses for which mother reported seeking care from:	199			186		
Any provider	157	78.9	[72.7–84.0]	124	66.7	[59.6–73.1]
Skilled provider^b^	151	75.9	[69.5–81.3]	116	62.4	[55.2–69.0]
> 1 provider	9	4.5	[2.4–8.5]	5	2.7	[1.1–6.3]
Proportion of children that sought care from category of provider^c**3**^**:**	199			186		
Govt hospital	0	0	–	5	2.7	[0.9–6.2]
Govt health center/post	122	61.3	[54.2–68.1]	111	59.7	[52.3–66.8]
Govt CBA/fieldworker	36	18.1	[13.0–24.2]	1	0.5	[0.0–3.0]
Pvt hospital/clinic	0	0	–	1	0.5	[0.0–3.0]
Pharmacy	1	0.5	[0.0–2.8]	2	1.1	[0.1–3.8]
Shop/market	2	1	[0.1–3.6]	9	4.8	[2.2–9.0]
Traditional/faith-based practitioner	5	2.5	[0.8–5.8]	0	0	–

^a^ARI defined as cough with chest-related difficulty breathing

^b^Skilled providers included government and private health facilities and government CBAs

^c^Calculated among all sick children—some children taken to multiple sources of care

**Fig. 2:**
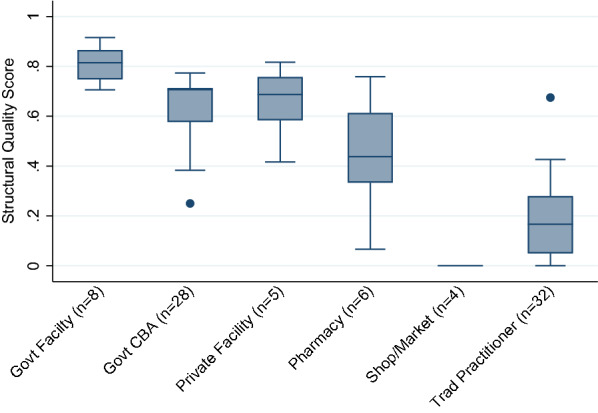
Median and interquartile range (IQR) of structural quality scores by provider category

Most skilled providers offered moderate to high levels of structural quality for managing child illnesses. Figure 2 presents structural quality scores by provider category. Structural quality scores varied most by category of provider, and in most cases did not vary greatly within provider categories used in the geographic linking. While there were a few providers whose scores were notably above or below others within their category, these were provider categories that were uncommon sources of care such as pharmacies and traditional practitioners. A detailed description of scores by provider type is available in a previous publication [6].

### Linking

**Fig. 3:**
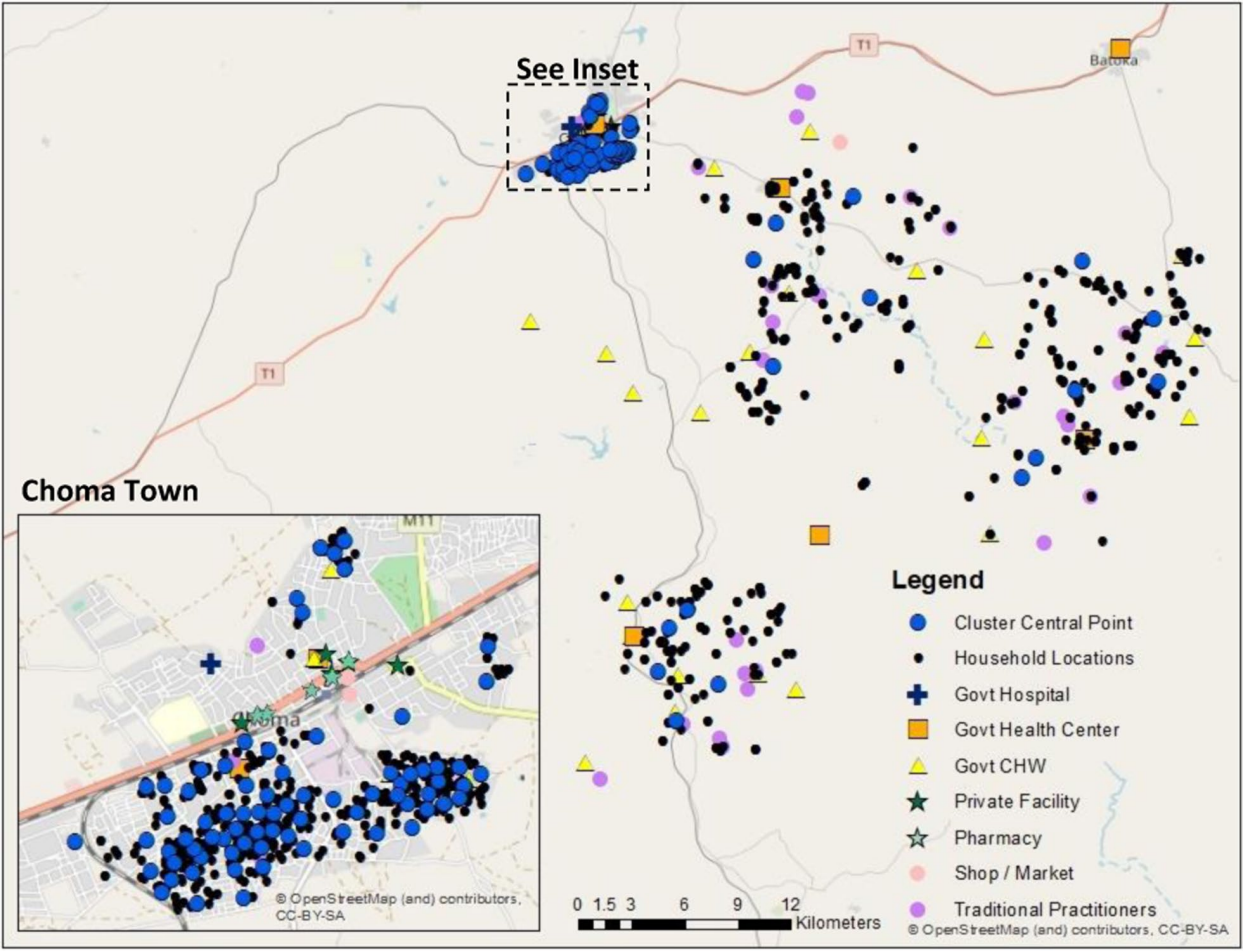
Map of cluster central points, households, and providers

Data on the exact reported source of care were available for 99% of rural care-seeking events and 93% of urban care-seeking events (Table 2). In the rural areas, we observed a greater distance in the shift from household location to central point location due to the low density of households in these areas requiring a greater geographic catchment to generate clusters of 150 households (Fig. 3). Using the household location, cluster central point, and displaced central point locations, we were able to link all children to a provider within the reported category of care using both the Euclidean distance and travel time methods as neither method capped the maximum distance to link to a provider. All urban children were linked to a provider within the reported care category using the 5 km radius method, however only 63.8%, 81.3%, and 47.6–72.9% of rural children linked to any provider using household, cluster central point, or displaced cluster location, respectively.

**Table 2:** Percent children linked to any provider within care category by method and stratum

	Rural %	Urban %
Exact-match	98.8	93
Euclidean distance		
HH location	100	100
EA central point	100	100
EA displaced A	100	100
EA displaced B	100	100
EA displaced C	100	100
EA displaced D	100	100
EA displaced E	100	100
Travel time		
HH location	100	100
EA central point	100	100
EA displaced A	100	100
EA displaced B	100	100
EA displaced C	100	100
EA displaced D	100	100
EA displaced E	100	100
5 km radius buffer		
HH location	63.8	100
EA central point	81.3	100
EA displaced A	59.0	100
EA displaced B	72.9	100
EA displaced C	47.6	100
EA displaced D	70.5	100
EA displaced E	69.3	100

Using household location, 89% of rural and 88.3% of urban children linked to their true reported source of care using Euclidean distance (Table 3). A lower proportion (73.2% rural; 84.5% urban) of children were linked to their true source of care when cluster central point location was used in place of household location. The proportion linked to their true source when using the displaced central point location ranged from 66.3 to 74.1% among rural children and 72.9 to 79.2% among urban children.

**Table. 3:** Percent children linked to true source of care by method and stratum

	Rural %	Urban %
Euclidean distance		
HH location	89	88.3
EA central point	73.2	84.5
EA displaced A	74.1	72.9
EA displaced B	66.3	74.4
EA displaced C	71.7	76
EA displaced D	67.5	76.7
EA displaced E	72.9	79.1
Travel time		
HH location	78	76.7
EA central point	56.1	45.8
EA displaced A	57.2	35.7
EA displaced B	64.5	46.5
EA displaced C	41	45.7
EA displaced D	54.8	35.7
EA displaced E	71.7	27.9

Compared to Euclidean distance, a lower proportion (78% rural; 76.7% urban) of children linked to their true reported source of care using travel time from household location (Table 3). The proportion linked to their true source of care fell to 56.1% of rural and 45.8% of urban children when using cluster central point location. The proportion linked to their true source when using the displaced central point location ranged from 41 to 64.5% among rural children and 27.9 to 46.5% among urban children.

### Effective coverage estimates

**Fig. 4:**
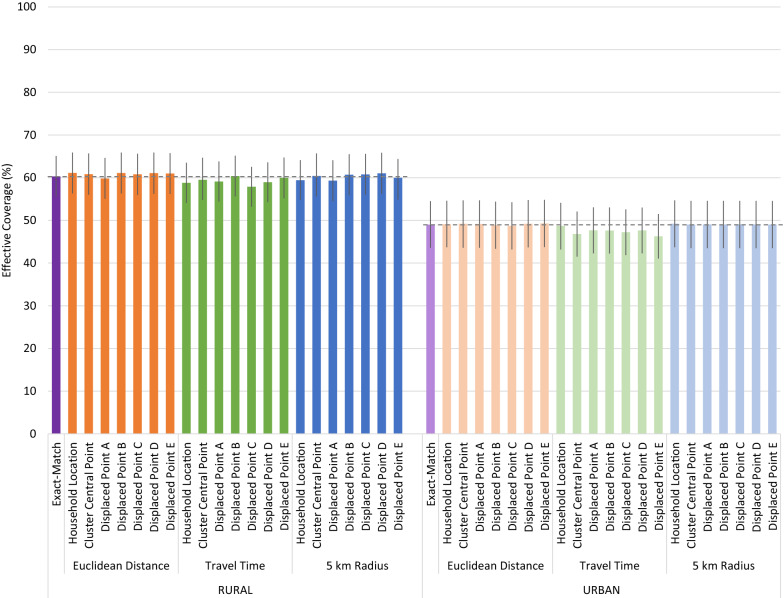
Effective coverage of management of child illness by linking method and child location

Despite the low to moderate proportion of children who linked to their true source of care using cluster central point and displaced cluster locations, all geolinking methods produced similar quality-adjusted effective coverage estimates compared to the precise exact-match method which assigned children to their true source of care (Fig. 4). Differences in quality-adjusted coverage estimated using the Euclidean distance and 5 km radius geolinking methods with varying underlying location data were minor and inconsistently under- and over-estimated the exact-match effective coverage estimates. In both the rural and urban strata, the travel time approach produced consistently lower quality-adjusted coverage point estimates across locations, but they were not statistically different from the exact-match or other geolinked estimates. None of the estimates generated using the central point location or displaced central point location were statistically different from the estimates generated using the specific household location.

### Sensitivity analysis

#### Quality simulation 1: preferential care-seeking from higher-quality facilities

**Fig. 5:**
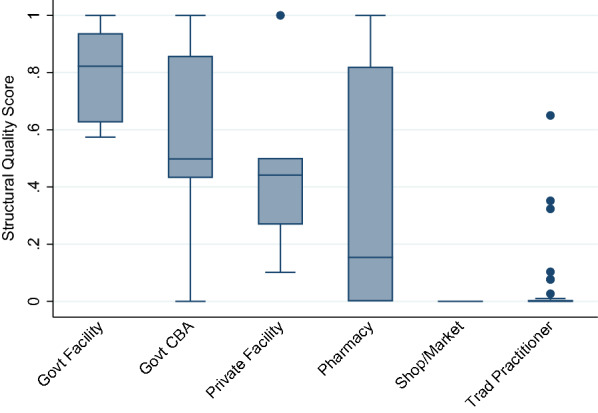
Median and IQR of structural quality scores by provider category, simulated preferential care-seeking from higher-quality providers

After simulating preferential care-seeking from higher quality providers using the original data set, we see a greater spread in quality scores within provider categories. We increased the scores of the more heavily utilized facilities, while less used facilities have reduced scores. The interquartile range (IQR) for the most common sources of care increased greatly (Fig. 5). The IQR increased by approximately 20 and 25 absolute percentage points from the original data for government facilities and CBAs, respectively.

**Fig. 6:**
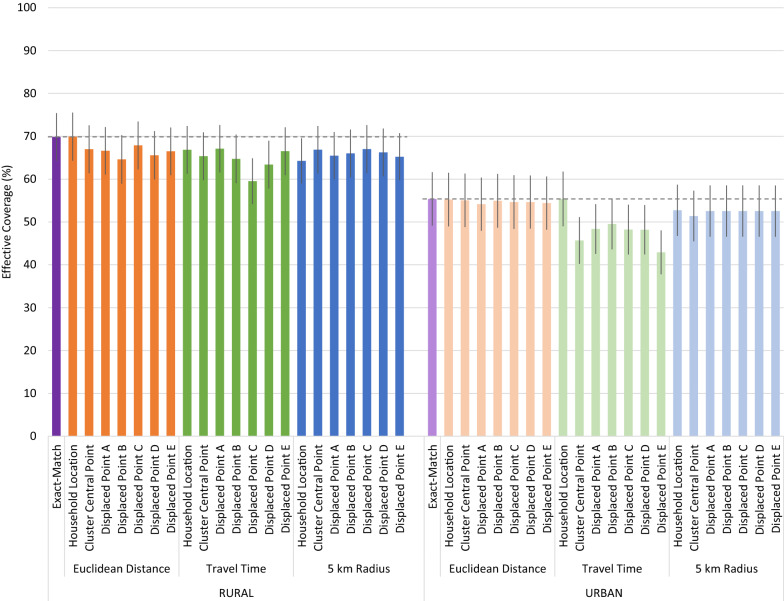
Effective coverage of management of child illness by linking method and child location, simulated preferential care-seeking from higher-quality providers

The simulated preferential care-seeking sensitivity analysis resulted in more pronounced deviations in quality-adjusted effective coverage estimates, notably in coverage estimates derived using travel time in the urban area (Fig. 6). The same random cluster displacements were used in the sensitivity analysis. Greater variability in provider quality within a level of care resulted in greater variability in effective coverage estimates using the travel time method. Estimates produced using the travel time method with household locations aligned closely with the exact-match method. However, the use of the cluster central point resulted in a significantly lower estimate of effective coverage than using household locations. Displacement of the central location did not significantly alter the coverage estimates when compared to the estimate generated using the undisplaced central point.

Some variability in scores produced using displaced central points with both the travel time and 5 km radius approach occurred in the rural area. However, only one of the estimates deviated significantly from the estimates generated using the geolinking approach with either the household or undisplaced central point.

#### Quality simulation 2: random quality

**Fig. 7:**
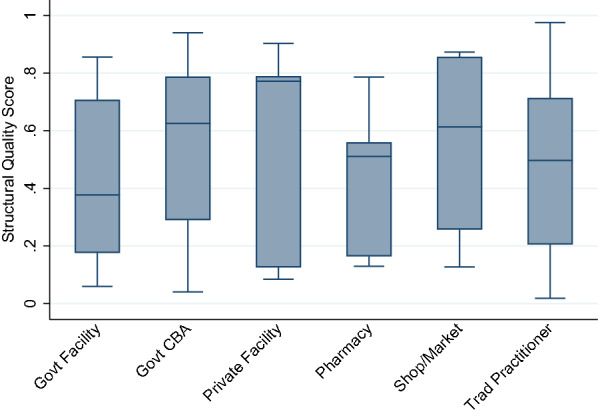
Median and IQR of structural quality scores by provider category, simulated random quality

Assigning each provider a quality score at random, we estimated the effect of imprecise location data on effective coverage estimates in settings of high variability in provider quality. Figure 7 shows the distribution of quality scores with random quality assignment. As expected, the median scores across provider types were approximately 50%, with an interquartile range of roughly 25 to 75%.

**Fig. 8:**
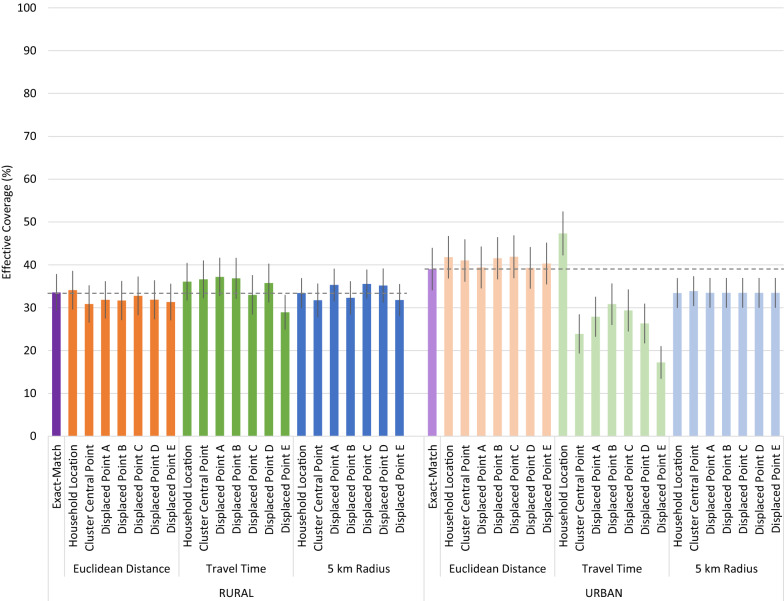
Effective coverage of management of child illness by linking method and child location, simulated random quality

As with the primary analysis and preferential care-seeking simulation, application of the travel time approach using randomly generated provider quality scores in the urban area resulted in the greatest variability in quality-adjusted effective coverage scores (Fig. 8). Calculation of shortest travel time from the central point linked more individuals to lower-quality providers than when the linking was performed using true household locations. In the urban area, the travel time linking approach over-estimated exact match effective coverage when using household locations and under-estimated effective coverage when using undisplaced and displaced central points.

In the urban area, averaging provider scores using the 5 km method resulted in a lower estimate of effective coverage compared to the exact-match method. The density of households resulted in the 5 km radius around either individual households or a central point encompassing most local providers. Slight variations in the central point of the buffer did not significantly alter the providers included in the aggregate score used in the effective coverage estimation, resulting in near-identical estimates regardless of location data used.

More variability in the effective coverage scores were apparent in the rural area using both the travel time and 5 km radius method. Due to greater geographic spread between providers within the rural area, there is greater variation in providers encompassed in the 5 km catchments than in the urban area. However, the effective coverage estimates produced using the 5 km radius method did not vary significantly from the exact match method or 5 km method using household location. Using the travel time method in the rural area, one of the five displaced central location produced an effective coverage estimate that differed significantly from the exact match and household location estimates.

## Discussion

We found that ecological linking based on geographic proximity accurately linked children to their true source(s) of care in most cases in this rural sub-Saharan African setting. However, using cluster central point location as a proxy for household location increased the proportion of children assigned to incorrect sources of care. Displacement of those central points variably increased and decreased the proportion linked to their true source of care. Despite this limited accuracy in identifying the true source(s) of care, estimates of quality-adjusted effective coverage of management of child illness generated using the ecological linking methods and central point locations did not differ significantly from estimates generated using data on the true source of care. The primary reason for the lack of effect on quality-adjusted coverage estimates may have been the limited variation in structural quality within key categories of providers.

In our geographic linking, we restricted the linked provider options to only providers within the source of care category reported in the household survey. This restriction meant that although children may not have been linked to their exact source of care, they were linked to a provider of the same level and managing authority. Within those provider categories that were most commonly utilized by the study population, namely government health facilities and CBAs, structural quality was reasonably consistent. As a result, a child could be linked to any provider within those categories and would experience a similar level of structural quality.

When simulating greater variability in provider quality, the choice of linking method and precision of the household location had a greater influence on effective coverage estimates. The 5 km radius method, which assigns each sick child the aggregate score of all providers (within the reported source of care category) within the 5 km radius, deviated significantly from the exact-match method when using randomly assigned provider quality scores in the urban area. However, this deviation was not due to the use of imprecise household location data as the estimates produced using the method applied to household locations differed from the exact-match estimate by a similar degree. The travel time method was most sensitive to shifts in the GPS coordinates used in the linking. Fewer children were linked to their true source of care using the travel time method, compared to the Euclidean distance method, and use of undisplaced or displaced central points further reduced the number linked to true source of care (28–72%) compared to using household location (rural: 78%, urban: 76.7%). When applying this method to a data set with greater variability in provider scores within a provider category, estimates of effective coverage deviated significantly from both the exact-match estimates and the estimates produced using travel time from household locations. As the Euclidean distance approach linked a majority (66–84%) of children to their true source of care when using undisplaced or displaced central points, this method showed the least variation in effective coverage scores calculated using central point locations.

This analysis was limited by its setting, which was characterized by relatively homogenous provider quality and care-seeking behavior. Although this health care landscape is similar to many sub-Saharan African settings [7], the sensitivity analysis demonstrates the conclusions of the primary analysis do not hold where care-seeking patterns are more diverse and provider quality is less consistent. Further, our measure of provider quality focused on structural factors and provider knowledge. It did not include gold-standard assessments of provider quality based on direct observation of care with clinical reassessment, which might have produced a more variable measure of provider quality in the primary analysis. Finally, data on enumeration area boundaries were unavailable. Using our census of household locations, we used geospatial analysis to derived clusters and cluster central points. This process created cluster central points that minimized the distance between household locations and each cluster central point. Our central points likely do not deviate as significantly from true household locations compared to central points in existing surveys that may use irregularly shaped enumeration areas and central point locations that do not align with true population density. As such, our analysis is likely a conservative estimate of the potential bias introduced through the use of imprecise household location data.

## Conclusions

This analysis suggests that use of displaced cluster central point location data in ecological linking analyses does not significantly bias measures of quality-adjusted effective coverage in settings where providers within the same general geographic area and provider category supply broadly consistent quality of care. However, it does provide evidence that using cluster central point or displaced data can introduce error when defining specific sources of care based on geographic proximity, even in settings where most children utilized the closest provider. Among the three geographic linking methods considered, linking by Euclidean distance consistently produced the least biased estimates of effective coverage. Caution should be used when interpreting measures of geographic proximity generated using non-specific location data such as cluster central points and displaced points.

## Data Availability

The datasets analyzed during the current study are available from the corresponding author on reasonable request. Household GIS data cannot be shared due to participant confidentiality concerns.

## Abbreviations

ARI: Acute respiratory infection.
CBA: Community-based agent.
DHS: Demographic and Health Survey
GIS: Geographic information system
GPS: Global positioning system.
MICS: Multiple Indicator Cluster Survey
SARA: Service Availability and Readiness Assessment
SPA: Service Provision Assessment
